# Factors Contributing to Rapid Early Infarct Expansion in Acute Ischemic Stroke Patients With Large Vessel Occlusion

**DOI:** 10.7759/cureus.59741

**Published:** 2024-05-06

**Authors:** Syed Mehmood Haider Abidi, Anoshia Arif, Ali Ahmed, Kanbar Fatima Zahra, Tauseef Raza, Salman Khan, Abdullah Imtiaz, Sabih Ahmed, Muhammad Ahsan, Muhammad Faiz Shad

**Affiliations:** 1 General Practice, Liaquat National Hospital, Karachi, PAK; 2 Medicine, Sindh Medical College, Jinnah Sindh Medical University, Karachi, PAK; 3 Medicine and Surgery, Sindh Medical College, Jinnah Sindh Medical University, Karachi, PAK; 4 Nephrology, Faculty of Medicine, Mohammed VI University of Health Sciences, Casablanca, MAR; 5 Orthopedics, Khyber Medical University Institute of Medical Sciences, Kohat, PAK; 6 Medical Unit, Divisional Headquarters Teaching Hospital/Gomal Medical College, Dera Ismail Khan, PAK; 7 Internal Medicine, Jinnah Postgraduate Medical Centre, Karachi, PAK; 8 Medicine, Abbasi Shaheed Hospital, Karachi, PAK

**Keywords:** vessel occlusion, acute ischemic stroke, rapid early infarct expansion, nihss, injury, ischemic, significance, hir

## Abstract

Background

Acute ischemic stroke, particularly in cases involving large vessel occlusion (LVO), poses a significant challenge due to the potential for rapid infarct expansion in the early phase. Such expansion, if not managed promptly, can lead to severe neurological deficits and poor clinical outcomes. Understanding the contributing factors that accelerate early infarct expansion is crucial for optimizing treatment strategies and improving patient prognosis. The main aim of the study is to determine the factors contributing to rapid early infarct expansion in acute ischemic stroke patients with LVO.

Methodology

The retrospective study was conducted at Liaquat National Hospital in Karachi from August 2023 to December 2023. Data were collected from 685 patients with anterior circulation LVO-related acute stroke with witnessed stroke onset and baseline perfusion imaging. Extracted clinical data included age, gender, medical history (hypertension, diabetes, etc.), and baseline National Institutes of Health Stroke Scale (NIHSS) scores.

Results

The mean age of the included patients was 67.4 years, with a relatively balanced gender distribution, i.e., 48.5% male (n = 332) and 51.5% female (n = 353). The mean baseline NIHSS score was 14.2, reflecting initial neurological severity. Imaging parameters revealed that 294 (42.6%) patients exhibited infarct expansion, with an average penumbra size of 23.5 mL. Hypoperfusion intensity ratio (HIR) quartiles demonstrated a notable association with progression rates, escalating from 27 (4%) patients in the first quartile to approximately 527 (77%) patients in the fourth quartile, highlighting a significant correlation between HIR and infarct expansion (p < 0.001).

Conclusions

HIR emerged as a pivotal factor strongly associated with rapid infarct expansion, underscoring its significance in predicting the trajectory of ischemic injury.

## Introduction

Acute ischemic stroke (AIS), particularly in cases involving large vessel occlusion (LVO), poses a significant challenge due to the potential for rapid infarct expansion in the early phase. Such expansion, if not managed promptly, can lead to severe neurological deficits and poor clinical outcomes [1]. Understanding the contributing factors that accelerate early infarct expansion is crucial for optimizing treatment strategies and improving patient prognosis. Identifying the factors that drive this rapid progression in ischemic stroke patients with LVO is essential for tailoring interventions aimed at mitigating infarct growth and preserving neurological function [2].

Mechanical thrombectomy is a superior approach for treating AIS caused by LVO in the anterior circulation compared to standard medical therapies [3]. The early identification of LVO proves pivotal in efficiently directing patients to specialized stroke centers, ensuring swift access to this effective intervention. To promptly recognize LVO, prehospital assessments encompass various components from the National Institutes of Health Stroke Scale (NIHSS) used by emergency medical services. Endovascular therapy (EVT) has emerged as a well-supported treatment for LVO, substantiated by evidence from five randomized trials [4]. The primary objective of EVT lies in impeding the expansion of the infarct core into the at-risk penumbra. Given the crucial influence of final infarct volume on outcomes, identifying factors that predict infarct growth is crucial in acute stroke management [5]. This study investigates the determinants associated with infarct growth among a cohort of EVT patients undergoing magnetic resonance imaging (MRI), recognizing that computed tomography (CT) remains the standard imaging modality for treatment selection, while diffusion-weighted imaging serves as a pivotal tool for measuring infarcted tissue volume in the hyperacute phase [6].

The primary objective of AIS treatment centers around restoring blood flow to the penumbra to rescue potentially viable tissue. Diverse hypoperfusion regions have been identified within the ischemic area early on, encompassing irreversibly damaged regions, at-risk yet viable tissue, and oligemic areas with no risk [7]. This affected tissue undergoes continual change, with the penumbra’s transformation into infarction, constituting a dynamic process. As time progresses, irreversible damage extends from the ischemic core toward the periphery. MRI assessments reinforce this idea by highlighting the varied destinies of the penumbra and the potential to detect the mismatch between irreversibly and reversibly damaged tissues [8]. Automated thresholding techniques enable real-time, accurate estimation of the penumbra using MRI or CT perfusion [9].

Objective

The main objective of this study was to determine the factors contributing to rapid early infarct expansion in AIS patients with LVO.

## Materials and methods

**Figure 1:**
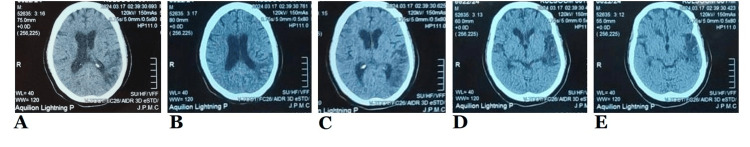
Computed tomography (CT) scans illustrating acute infarcts. A: An axial section of a plain CT scan of the brain showing a hypodense region indicative of ischemia on the left side of the cerebral hemisphere possibly due to middle cerebral artery (MCA) occlusion. B: An axial section of a plain CT scan of the brain showing a hypodense region indicative of ischemia on the left side of the parietal lobe possibly due to MCA occlusion. C: An axial section of a plain CT scan of the brain showing a hypodense region indicative of ischemia on the left side of the temporal lobe possibly due to MCA occlusion. D: An axial section of a plain CT scan of the brain showing ischemic changes on the left side of the brain parenchyma, a territory supplied by MCA. E: An axial section of a plain CT scan of the brain showing hypodense changes on the left side of the cerebral hemisphere possibly due to an infarct.

The retrospective study was conducted at Liaquat National Hospital in Karachi from August 2023 to December 2023. Data were collected from 685 patients with anterior circulation LVO-related acute stroke with witnessed stroke onset and baseline perfusion imaging (Figure 1).

Inclusion criteria

The study included confirmed cases of AIS with LVO involving patients aged 18 years and older. Inclusion criteria required the availability of baseline imaging, such as MRI or CT scans, for accurate assessment, and participants needed to present within a defined time frame from symptom onset, for example, within 24 hours.

Exclusion criteria

Exclusion criteria involved patients with stroke resulting from etiologies other than LVO. Additionally, individuals with a history of previous strokes were excluded to focus specifically on acute cases.

Data collection

The medical records of patients meeting the inclusion criteria were systematically identified from hospital databases or registries. Extracted clinical data included age, gender, medical history (hypertension, diabetes, etc.), and baseline NIHSS scores. Baseline imaging data from MRI or CT scans were reviewed to determine infarct expansion patterns, penumbra size, and the extent of ischemic damage. Furthermore, the time intervals from symptom onset to various key points, including imaging, were meticulously recorded for temporal assessments. The research team ensured adherence to ethical guidelines and patient confidentiality throughout the data collection process.

Statistical analysis

Data were analyzed using SPSS version 29.0 (IBM Corp., Armonk, NY, USA). The analysis aimed to discern correlations and identify significant factors contributing to rapid early infarct expansion in AIS patients with LVO, providing crucial insights into stroke management strategies. The chi-square test was done to assess the association between hypoperfusion intensity ratio (HIR) quartiles and progression rate. Pearson correlation analysis was employed to examine the associations between the baseline NIHSS score, time to imaging, and extent of ischemic damage with infarct expansion in the study. P-values <0.05 were considered significant.

Ethical considerations

Ethical approval was obtained from the Sindh Medical College, Jinnah Sindh Medical University, Karachi, Pakistan. Data were collected after informed consent was obtained from study participants.

## Results

The retrospective study conducted at Liaquat National Hospital in Karachi included 685 patients, with a mean age of 67.4 years. The gender distribution was relatively balanced, comprising 48.5% males (n = 332) and 51.5% females (n = 353). Pre-existing conditions were prevalent, with 62.3% of patients (n = 426) having hypertension, and 34.8% (n = 238) affected by diabetes. The mean baseline NIHSS score was 14.2, reflecting the initial neurological severity. Regarding imaging parameters, 42.6% of patients (n = 294) exhibited infarct expansion, with an average penumbra size of 23.5 mL. Regarding the extent of ischemic damage, 78.9% of the patients in the study exhibited moderate-to-severe ischemic damage, indicating a significant impact on their condition. This suggests a high prevalence of serious tissue damage due to inadequate blood supply. These values provide quantitative insights into patient demographics, clinical characteristics, and imaging findings, contributing valuable context to the study’s exploration of factors influencing rapid early infarct expansion in AIS patients with LVO (Table 1).

**Table 1:** Demographic data of patients. A high NIHSS score indicates more severe neurological impairment, while a low NIHSS score indicates less severe impairment. NIHSS = National Institutes of Health Stroke Scale

Characteristic	Value
Total patients	685
Mean age (years)	67.4
Gender distribution, N (%)	
Male	332 (48.5%)
Female	353 (51.5%)
Hypertension, N (%)	426 (62.3%)
Diabetes, N (%)	238 (34.8%)
Baseline NIHSS, mean	14.2
Imaging parameters	
Infarct expansion, N (%)	294 (42.6%)
Mean penumbra size (mL)	23.5
Extent of ischemic damage	Moderate to severe (78.9%)

Time intervals from symptom onset to imaging averaged 4.7 hours, while the duration from symptom onset to treatment averaged 6.2 hours (Table 2).

**Table 2:** Symptom onset to imaging and treatment.

Time intervals	Mean values
Symptom onset to imaging	4.7 hours
Symptom onset to treatment	6.2 hours

HIR quartiles demonstrated a notable association with progression rates, escalating from 4% in the first quartile to approximately 77% in the fourth quartile, highlighting a significant correlation between HIR and infarct expansion (p < 0.001) (Table 3).

**Table 3:** Association between HIR quartiles and progression rate. Chi-square test. HIR = hypoperfusion intensity ratio

HIR quartile	Progression rate	P-value
First quartile	4%	<0.001
Second quartile	~15%	<0.001
Third quartile	~50%	<0.001
Fourth quartile	~77%	<0.001

Higher baseline NIHSS scores demonstrated a strong positive correlation (r = 0.67, p < 0.001), signifying a substantial association between initial neurological severity and infarct expansion. Additionally, the time interval to imaging exhibited a moderate positive correlation (r = 0.42, p < 0.01), implying a relationship between delayed imaging and increased infarct expansion. The extent of ischemic damage displayed a robust positive correlation (r = 0.54, p < 0.001) (Table 4), highlighting the significant impact of the severity of initial damage on subsequent infarct expansion in these patients.

**Table 4:** Factors associated with infarct expansion. Pearson correlation analysis. NIHSS = National Institutes of Health Stroke Scale

Factors	Correlation (r)	P-value
Baseline NIHSS score	0.67	<0.001
Time to imaging	0.42	<0.01
Extent of ischemic damage	0.54	<0.001

## Discussion

This study examined the significance of the HIR as a critical component associated with fast infarct advancement in AIS with LVO. The prevalence of hypertension (62.3%) and diabetes (34.8%) in the patient cohort is consistent with the recognized association of these conditions with an increased risk of stroke [10]. The HIR emerges as a pivotal factor associated with rapid infarct progression in AIS with LVO, irrespective of the imaging technique employed [11-13]. These findings hold critical implications for the design of neuroprotection trials and for guiding triage decisions in primary stroke centers. Prompt identification of AIS with LVO is essential for effectively triaging patients eligible for mechanical thrombectomy, as earlier endovascular intervention has been linked to improved functional outcomes [14-16].

The mean baseline NIHSS score of 14.2 underscores the severity of neurological impairment in the studied population. Previous research has focused on predicting LVO using various scoring systems based on specific elements of the NIHSS owing to its accuracy in LVO prediction [17]. The observed infarct expansion in 42.6% of cases with an average penumbra size of 23.5 mL is consistent with studies linking larger penumbras to worse clinical outcomes and greater infarct expansion [18].

The study noted a moderate-to-severe extent of ischemic damage in 78.9% of cases. Efforts to simplify prehospital assessments have led to extensive exploration of modifications to the complex NIHSS. This study contributes by investigating various stroke-related factors to identify additional predictors of LVO in the anterior circulation. Our findings reveal independent associations of NIHSS, current smoking status, intracranial atherosclerotic disease, extracranial atherosclerotic carotid stenosis, and type 2 diabetes mellitus with anterior circulation LVO [19-22]. Notably, our developed model demonstrates higher accuracy in predicting LVO compared to using NIHSS alone [23]. This underscores the significance of incorporating past medical history into the predictive model, particularly within a healthcare system offering longitudinal patient care.

Study limitations

The retrospective study at Liaquat National Hospital in Karachi sought to determine the variables that lead to fast early infarct enlargement in AIS patients with major artery obstruction. Although the study emphasized that the HIR is a crucial component closely linked to infarct expansion, it has some shortcomings. The single-center and retrospective approaches might lead to selection bias and restrict the generalizability of the study findings. Emphasizing anterior circulation LVO-related strokes neglects possible knowledge of posterior circulation LVO instances. The study’s limited data collection timeframe and omission of specific variables may hinder a thorough comprehension of the factors influencing infarct expansion. The diversity in imaging methods and the lack of investigation into external variables affecting therapy initiation complicate data analysis. The study findings highlight the significance of HIR but stress the necessity of being cautious when extending conclusions outside the study’s particular setting and design limitations.

## Conclusions

HIR emerged as a pivotal factor strongly associated with rapid infarct expansion, underscoring its significance in predicting the trajectory of ischemic injury. These findings underscore the multifaceted nature of factors contributing to rapid early infarct expansion in LVO stroke and emphasize the importance of expedited intervention strategies. Understanding these determinants not only aids in risk stratification but also informs clinical interventions, potentially paving the way for more tailored and precise therapeutic approaches aimed at mitigating rapid infarct progression and improving patient outcomes.
